# Structural optimisation for controlled deflections of additively manufactured single material beams

**DOI:** 10.1038/s41598-023-33946-x

**Published:** 2023-04-28

**Authors:** Wuxin Yang, Malaya Prasad Behera, Yifan Lv, Loulin Huang, Sarat Singamneni

**Affiliations:** Auckland University of Technology, Auckland, New Zealand

**Keywords:** Engineering, Mechanical engineering, Materials science, Structural materials, Mechanical properties

## Abstract

Closely controlling the mechanical behaviour and characterization of the deflection of a beam structure is a well-known and widely studied engineering problem. The progress in additive manufacturing methods and the possibilities to closely control the material property variations with the controlled placement of materials further widen the opportunities to achieve given beam deflection criteria. The multi-material additive manufacturing solutions suffer from the lack of real engineering material options, and the quality and performance of the printed parts are usually unsuitable for producing functional parts. A novel cellular structured solution is proposed here, which utilises optimisation of geometries of individual cells of a single material structured beam to obtain deflection profiles closely matched with preset conditions under different loading conditions. The cellular geometry of the structured beam is continually altered for searching and converging on the optimal structure of the cells by the covariance matrix adaptation evolution strategy algorithm in an iterative manner. The optimised beam structures could also be physically produced with single material additive manufacturing methods and the experimental and numerical beam deflection responses correlated closely.

## Introduction

Compliant mechanisms have become to play significant roles in systems such as soft robots^1–3^, leaf springs^4,5^, and flexible hinges^6–8^, considering reduced friction, wear and tear, and the lack of the need for lubrication and assembly, compared to the rigid-body solutions^6^. First proposed by Her and Midha in the 1980s^9^, theoretically, these are mechanical devices gaining their mobility through the elastic deformation of flexible parts^10^. Deflections of mechanical elements such as beams are used to transfer force and motion without the need for movable parts, joints, and other driving mechanisms and find applications in robotic leg mechanisms^1–3,11^ and grippers^12–14^. Leaf springs that use elastic deflection to absorb and store the energy of impacts and flexible hinges are also widely used as compliant mechanisms that gain mobility while eliminating the sliding friction of contact surfaces^7^. In any case, precisely controlling the elastic deflection in the compliant system becomes an essential design factor for accurate grip and gait simulations and other responses.

The effective application of the compliant mechanisms, therefore, relies on the closely controlled mechanical behaviour of beam structures. The prediction and characterisation of the beam deflection mechanics is a well-known and widely studied engineering problem as the evaluation of the deflection curves of simply supported or cantilever beams under different loading conditions traces back to Galileo since 1638^15^. Numerous solutions have evolved ever since, such as double integration, moment area, and finite element methods^6,16^ to evaluate the beam deflections closely. Altering the beam structural form to comply with preset deflection or other response patterns needs more advanced analysis tools, though, where multi-objective optimisation and numerical simulation of continuum mechanics often come together.

For example, the solid isotropic material with the penalisation (SIMP) method^17^ uses material density optimization to identify whether each element is solid or void in a fixed domain of finite elements to achieve topology optimisation^18^. SIMP has been applied to beam structural optimisation problems to achieve outcomes such as optimal stiffness-to-weight ratios^19–21^. The truss-based ground structure method can control the mechanical properties of the structure by optimising the elements, the number of nodes, and the truss locations^22^. Ground structure methods have been applied to optimal design for minimum compliance-and-weight^23^ and weight minimisation with stress and displacement limitations^24^. The Particle Swarm Optimization (PSO) algorithm has also been proposed to achieve desired beam deflection curves by altering the widths of the beam segments^25^. The bi-directional evolutionary structural optimisation (BESO) method is also well studied for improving the mechanical properties of beams, allowing the object to evolve to the optimal structure by gradually removing the redundant material and adding material to locations of high sensitivity^26^. BESO has been used for optimising various target outcomes such as stiffness-to-weight ratio^27–29^, beam geometry under multiple load cases^30^, shell-infill designs for optimal stiffness and robust performance^31^, and multi-objective stiffness and fundamental natural frequencies^32^. The evolutionary non-dominated sorting genetic algorithm II (NSGA-II) was used for optimising the beam geometry for desired deformation behaviour^33^. Other methods, such as the level-set method and compositional pattern-producing network (CPPN) also been adapted to optimise the lattice mesostructures for superior stiffness-to-weight performance^34,35^.

While optimisation of structural forms based on single materials has seen considerable advances in both mathematical and computational evaluations, topology optimisation of multi-material domains would offer a plethora of new opportunities. The limitations of traditional manufacturing methods restrict structural forms to be of single material domains, but the advances in multi-material additive manufacturing have opened up new avenues to consider several multi-material based topology optimisation schemes targeting optimal mechanical property responses of beams. Gradual BESO, an enhanced version of BESO, capable of handling multiple-material and geometry cases, was proposed for stiffness-to-weight ratio optimisation^36^. Genetic algorithms (GA) were also adopted to optimise the distribution topology of soft and stiff materials within the beam domains for desired deformation curves and other responses^34,37^. However, multi-material approaches usually suffer from the lack of real engineering material options. Current multi-material additive manufacturing systems can only offer digitally mixed acrylic polymer options consolidated with UV curing, with limited potential in real world engineering applications^38^. The properties are not comparable to real engineering polymers such as polyethene, polypropylene, Polystyrene, ABS, Nylon, etc., commonly used for engineering applications and processed by injection moulding traditionally or by FDM and SLS in the additive manner.

A novel alternative is proposed in this paper which brings the benefits of both additive material consolidation and multi-objective optimisation schemes together to achieve topology optimisation of single material beam structures to match with the preset deflection responses under multiple loading conditions. The problem domain is first discretised into a number of volume elements or voxels. Instead of using solid voxels, hollow brick-like elements are used, where, by controlling certain critical dimensions, the effective mechanical properties and mass of each element can be controlled. Based on these structured voxels, the mechanical properties of the structural domain is governed by the properties of the single material and the overall geometry, as well as the internal cellular structure of each voxel, while preserving the original overall geometry. Such macro changes in the structures of voxels are possible to achieve in real engineering materials by additive manufacturing technologies, such as fused deposition modelling and selective laser sintering and melting, that have become common in recent years. For searching the generative design spaces, several algorithms were considered, such as Covariance Matrix Adaptation Evolution Strategy (CMA-ES), GA, SIMP and PSO. A detailed comparison of the relative merits of different search algorithms is summarised in Table 1. However, compared to the other solid-or-void optimisation methods, CMA-ES is a natural choice which can continuously vary the parameter with adjustable step sizes. Therefore, the Covariance Matrix Adaptation Evolution Strategy (CMA-ES) method was used in this work to optimise the beam structure to match with the preset deflection responses by varying the voxel geometries.

**Table 1:** A summary of the comparison between the proposed method and other possible optimisation methods.

Structural optimization algorithms	SIMP	PSO	ESO	BESO	GA	CPPN
Lack of capabilities	Solid-or-void method	Adjustable step size. But computationally costly	Solid-or-void method	Solid-or-void method	The solid-or-void method with a fixed step size and computationally costly	Pattern-based solid-or-void method. Less flexibility for numerical optimisation
Proposed method	Adjustable step size with more degrees of freedom	Less computational cost	Adjustable step size with more degrees of freedom	Adjustable step size with more degrees of freedom	Adjustable step size with more degrees of freedom	Flexible for numerical value optimisation

The resulting overall optimised structure geometry can be fabricated with a single material using either selective laser sintering or melting, depending on whether polymeric or metallic materials are opted for. CMA-ES was originally designed and developed for handling optimisation problems involving continuous variation of the critical parameters^39^. It has been widely used in various studies in computer science and engineering, such as parameter tuning for neural networks and ranking support for vector machines^40–43^. Truss layout optimisation for minimising the weight^44^, topology optimization for cloaking polarized light^45^, and optimising metallic shapes and dielectric properties for desired electromagnetic scattering and radiation characteristics^46^ are other examples of applications. However, there was no evidence that the generative design method CMA-ES was adopted for controlling beam deflection behaviour by structural optimisation. The proposed approach fills this gap, demonstrating the ability to control the beam deflection behaviour under multiple loading conditions by cellular structural optimisation using CMA-ES. The research focus is not the comparative evaluation of different optimisation algorithms. The key research question is whether single material lattice structures can be optimised by using one of the generative design schemes to produce beam structures that can be additively manufactured readily with a single material and at the same time confining to pre-defined deflection patterns. Analytical and numerical modelling outputs, experimental results and validation are elaborated in “Results and discussion” section, and the key conclusions drawn based on the inferences from the research results are briefed in “Conclusion” section.

## Methodology

### Generative design and experimental validation

Generative design represents the design discipline that applies the natural inspiration of variation and reproduction of all life. The "survival of the fittest" mechanism enforces the reproduction of each generation to move towards targets specified by the objective function in an iterative manner. Then the "survivors" can be selected from the final generation^47^. The proposed approach is to vary the distribution of effective stiffnesses and density by altering the structure of the voxels while the base material and the overall geometry remain the same. With the advent of increased computational power and improved algorithms, generative design methods have been applied to finding solutions in numerous computer science and engineering applications^48,49^. For the current problem, the method is to locally alter the internal structure of the voxels to achieve the variation of the material property while keeping the overall geometry the same. The problem domain is divided into multiple brick-like hexahedral elements or voxels with the same length and width. The structural parameter of the voxels, such as the sizes, numbers and the location of the internal hollow structures, are represented as numerical parameter inputs for the optimization algorithm. A standard three-point bending test is selected to experimentally verify and compare the actual deflection behaviour of the optimised structures. The generative design algorithm and methodology are discussed in “Methodology” section. The beam problem case, cellular geometries, boundary limits, material properties, and preset deflection conditions are presented in “The beam deflection case study” section.

### Optimisation algorithm and functional evaluation

**Figure 1:**
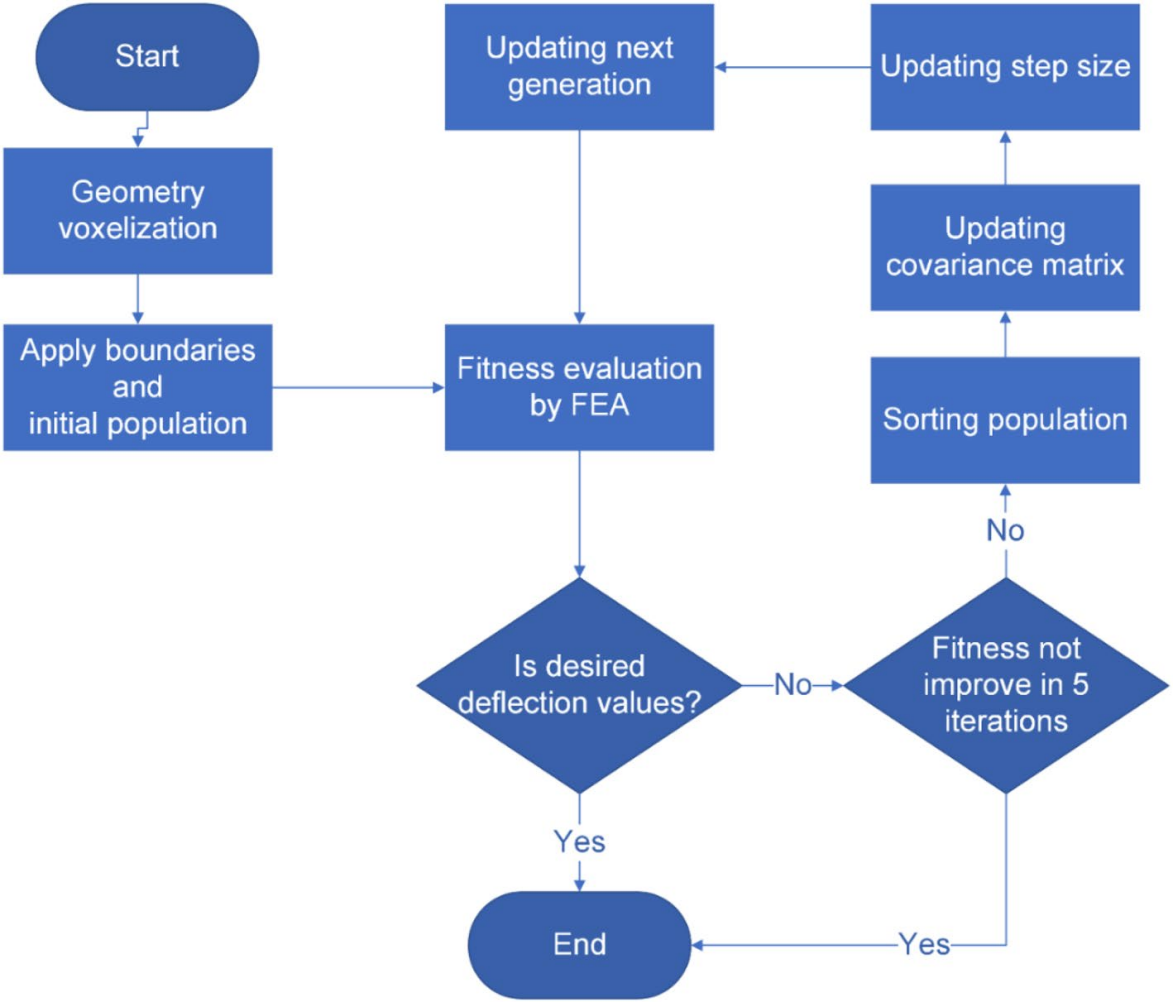
The flowchart of the overall optimisation scheme based on the implementation of the CMA-ES algorithm with MATLAB and Comsol Multiphysics solution for the beam deflection problem.

The covariance matrix adaptation evolution strategy (CMA-ES) algorithm is designed for handling continuous optimisation problems^39^. Studies claim that CMA-ES gives superior performance amongst more than a hundred classical and modern optimisation algorithms with different black-box functions^50^. In this work, the CMA-ES algorithm originally proposed by Hansen and Ostermeier^39^ is adopted to optimise the geometries of cellular structures, targeting the convergence of the beam deflection to a pre-defined form. CMA-ES is an iterative algorithm. Firstly, the initial population is created by randomly varying the void size within the geometric boundaries as defined by the accuracy limitations of the 3D printing system. Then, in each iteration, the offspring or a number of candidate solutions for the next generation are sampled from a normal distribution of multiple variables with a step size of the current generation. The fitness of these solutions is evaluated, and the sampling distribution of the next generation is adjusted according to the fitness of each solution in the current generation^39^. Then, the covariance matrix for the next iteration is updated based on the gradient of the improvement of the previous iteration. Finally, the step sizes for the next iteration are calculated based on the current covariance matrix of the fitness value. The iterative analysis will be terminated when the desired deflection values set as target sets are reached. The major steps of the structural optimization scheme for searching the optimal geometry with preset deflection profiles are summarized in the flowchart, as shown in Fig. 1.

Numerical modelling methods have been widely used for predicting and improving the AM processes. Different modelling approaches have been developed for handling various AM processes and their key processing parameters for improving the printing quality and design flow^51–53^. For example, for a better understanding of the residual stresses and distortions during the AM processes, the thermal history of the manufacturing procedure can be modelled for the part quality control^54^, as well as, the relation between thermal stress and scanning strategies^55^.

For the modelling part of this research, the commercial finite element analysis software package COMSOL Multiphysics (Version 5.6, release date November 11, 2020, COMSOL Inc., Stockholm, Sweden) is used for evaluating the deflection behaviour. The standard quadratic solid elements are used for finite element discretisation and analysis. The objective function of the fitness value is defined as the weighted (*w*) mean square errors of the actual and the target deflection of each selected point (*i*) in the structure. The reason for the use of weighted mean squares is that depending on the load and geometry settings, large differences in deflection values may result, which need to be considered with proper weighting. The overall optimisation scheme is implemented in MATLAB (R2017B, release date September 14, 2017, MathWorks, Natick, Massachusetts, USA)

1 $$fitness = \mathop \sum \limits_{i = 1}^{n} w_{i} \left( {deflection_{i}^{t\arg et} - deflection_{i}^{result} } \right)^{2}$$

2 $$w_{i} = l^{2} /d_{i}$$

where $deflection_{i}^{t\arg et}$ and $deflection_{i}^{result}$ are the target and the deflection computed by the finite element analysis; $n$ and $i$ indicate the total number of the sampling point and the index of the deflection of the current sampling point. The weight value is calculated by the length of the beam $l$ and the distance between the points of load and the closest supporting points $d$.

## The beam deflection case study

### Voxelization and cellular geometry settings

**Figure 2:**
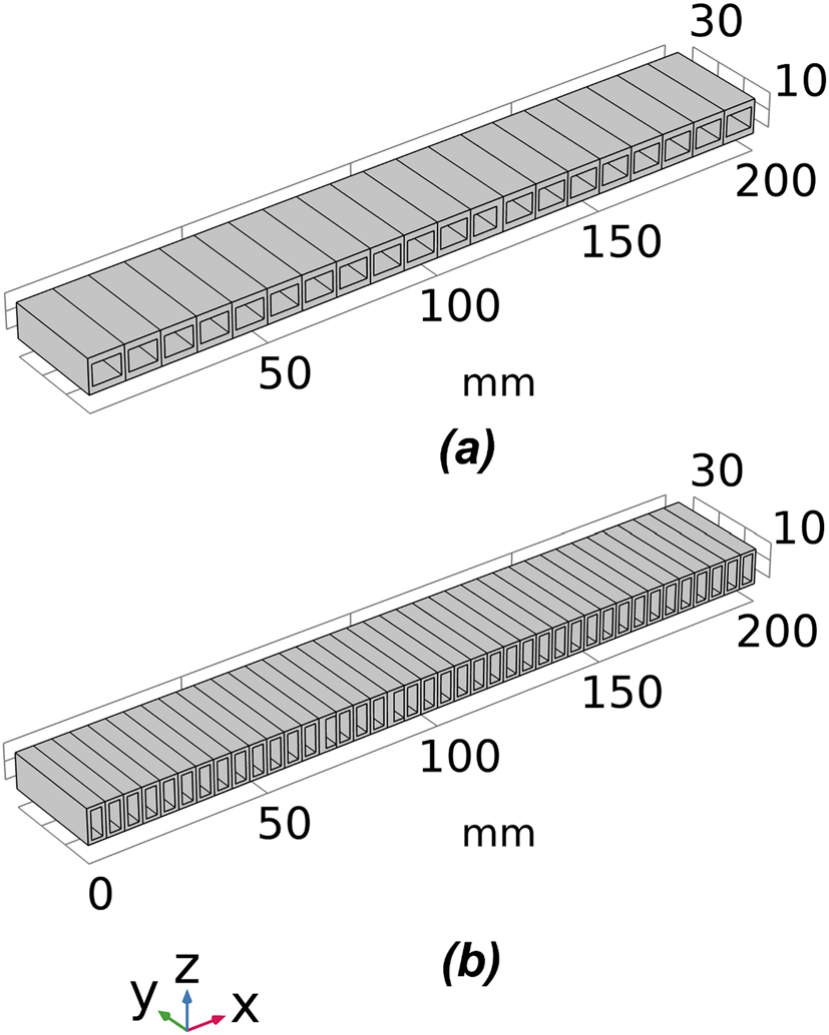
The cellular beam structures with lengths of voxels 10 mm and 5 mm, respectively, for (**a**) 20 voxel and (**b**) 40 voxel models *(Images are created by the authors using Solidworks Educational Version 30.3.1.2 2022-23).*

**Figure 3:**
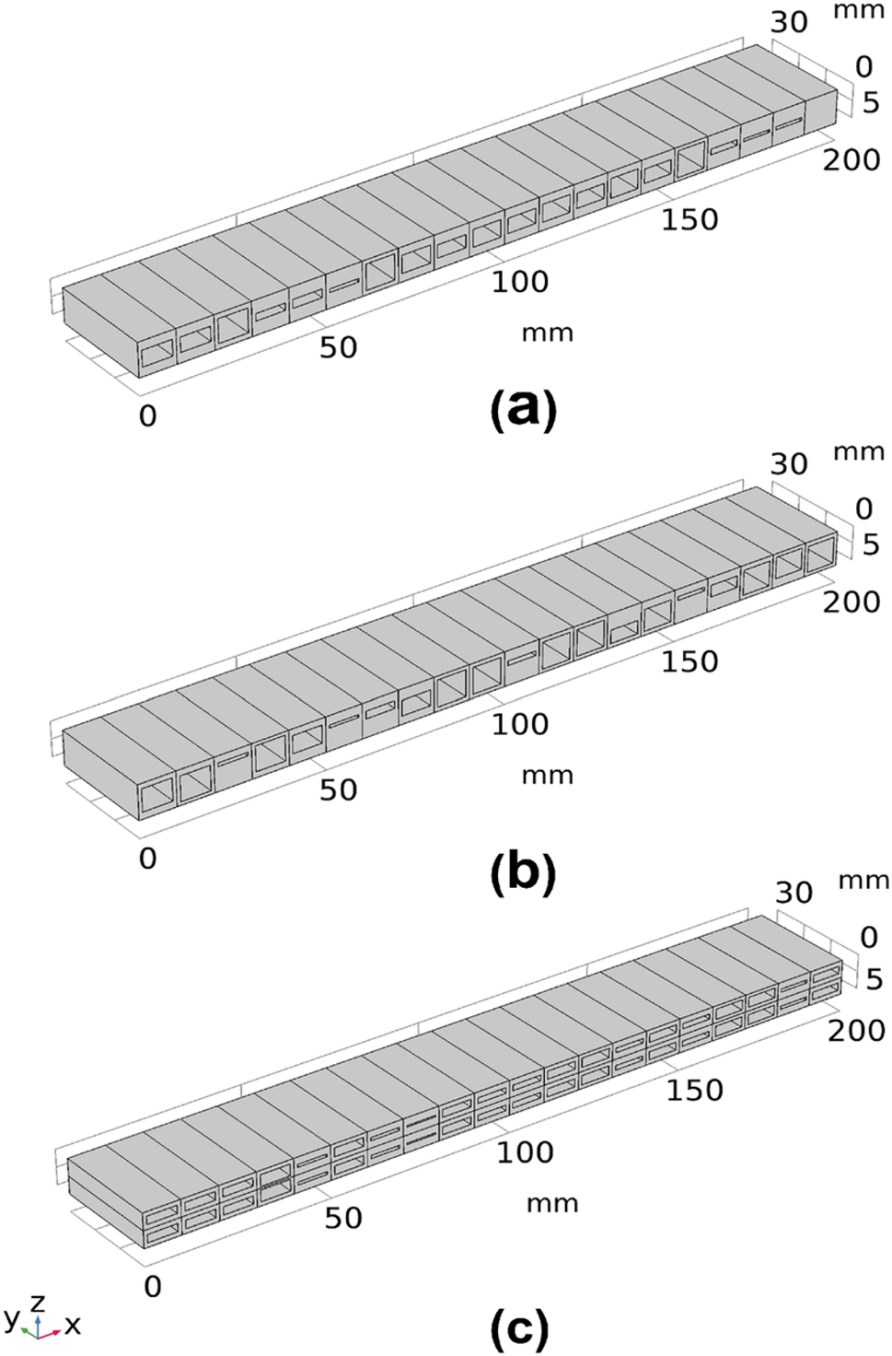
CAD models of the 20 voxel structures with the three different voxel cellular geometry settings; (**a**) single symmetric void, (**b**) single asymmetric void and (**c**) double void settings. *(Images are created by the authors using Solidworks Educational Version 30.3.1.2 2022-23).*

Two setups of voxelisation are tested, which divided the beam structure into 20 and 40 voxels, respectively. The overall dimensions (L × B × H) for the beam structure remain the same at 200 mm × 30 mm × 10 mm for all voxelisation and cellular structure settings. The CAD models of the beam structures with different voxelisation subdivisions are shown in Fig. 2. The CMA-ES optimisation algorithm will alter the height of the void, which changes the moment of inertia of each voxel and controls the overall deflection behaviour to match with the desired deflection profile. Further, three voxel geometry settings are designed to comprehensively validate the performance of the proposed optimisation method, namely, symmetric and asymmetric single void and double void voxel geometry settings. The difference between symmetric and asymmetric geometries is that the voids are centrally placed in the former while they are asymmetric to the central line in the later. CAD models of the three voxel geometry settings are depicted in Fig. 3.

**Figure 4:**
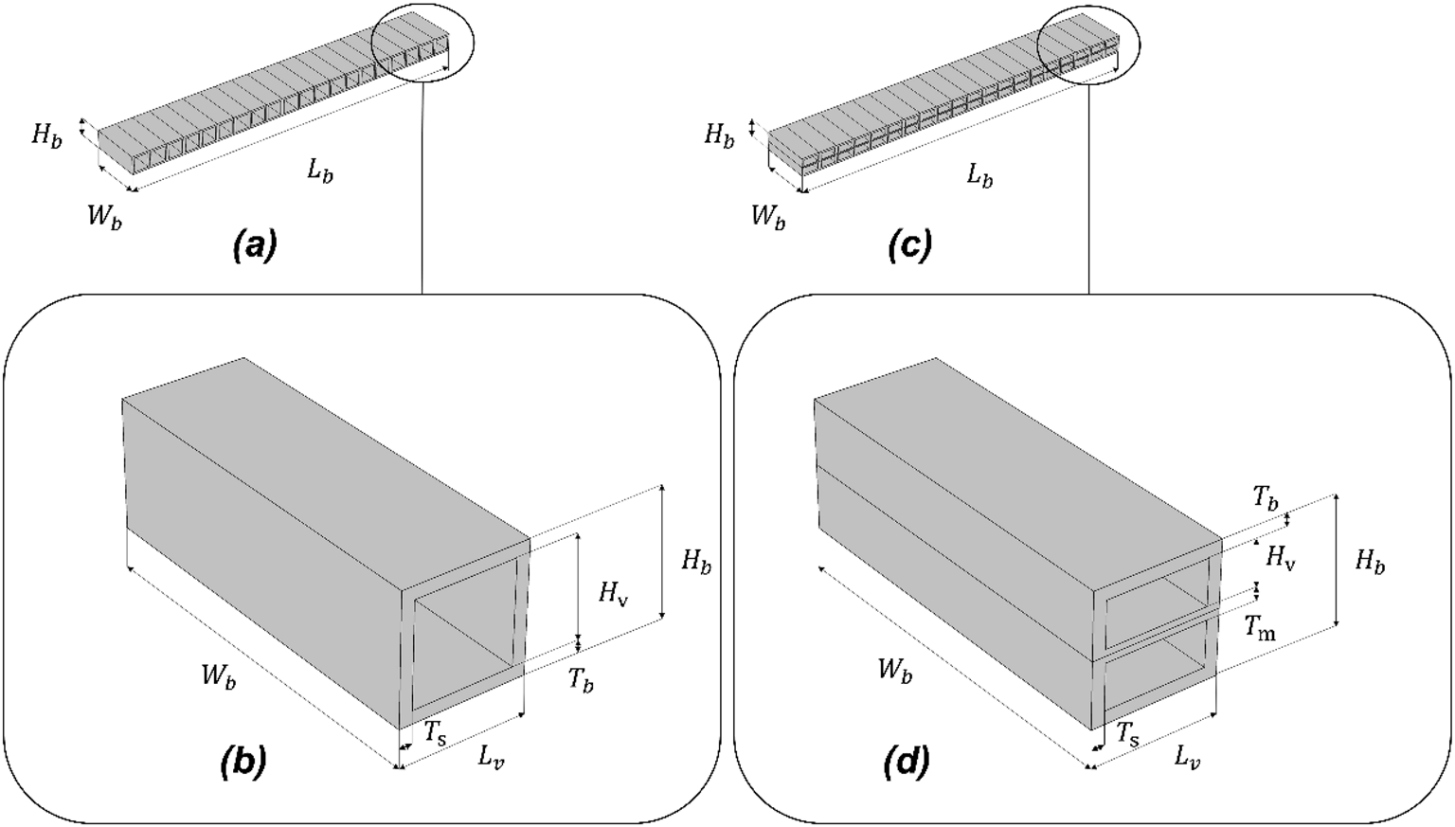
The beam domain example for 20 voxels model with single symmetric or asymmetric void setting (**a**) and double void setting (**c**). The details of voxel geometry settings are shown in (**b**,**d**), respectively. In these particular examples, the voxel height $H_{b}$ is 10 mm for both single and double void settings (**b**,**d**). The hollow structure's height $H_{v}$ is 8 mm for single void model in (**b**) and $H_{v}$ is 4 mm for double void model in (**d**). *(Images are created by the authors using Solidworks Educational Version 30.3.1.2 2022-23).*

In order to maintain the overall dimensions of the beam structure the same, the height of the hollow structure of each voxel ($H_{v}$) is used as the optimisation parameter for both symmetric single void and double void geometries. An additional optimisation parameter $T_{m}$ is used to control the location of the void within the voxel. The detailed voxel geometry settings are depicted in Fig. 4

### Boundary settings for the cell geometry

The limits of the optimisation parameters are related to practical considerations such as the accuracy and the maximum build dimensions of the AM equipment readily available at the Additive Manufacturing Research Centre of the Auckland University of Technology. Considering the fabrication quality and maintaining the overall dimensions, the height of the beam ($H_{b}$) is fixed at 10 mm, and the minimum thickness is limited to 1 mm. As a result, the minimum top and bottom flange thickness $T_{b}$ and the side flange thickness $T_{s}$ of each voxel are set to be equal to 1 mm. For the double void voxel geometry, the minimum thickness of the middle flange $T_{m}$ is also limited to 1 mm. With these as the limiting boundary values, the single void voxel structure (for both symmetric and asymmetric settings) can vary anywhere from a solid cell to a voxel with a hollow structure of 8 mm height $H_{v}$.

**Figure 5:**
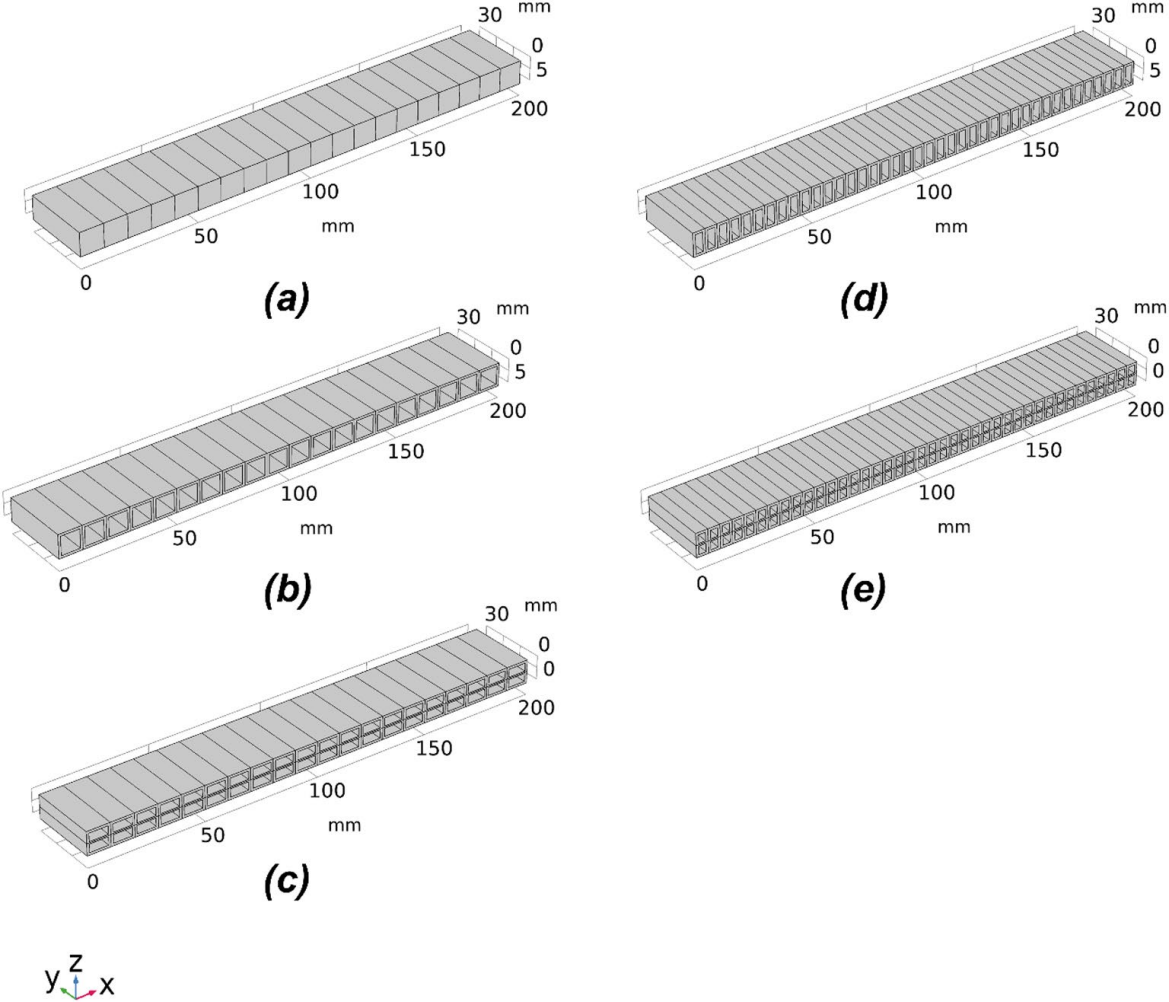
The low bound and high bound structural settings for the voxels for optimising the deflection behaviour of the beam. (**a**) the low bound structure for all voxelisation settings; (**b**) high bound structure for 20 voxels model with single symmetric or asymmetric voids; (**c**) high bound structure for 20 voxels model with double voids; (**d**) high bound structure for 40 voxels model with single symmetric or asymmetric voids; (**e**) high bound structure for the 40 voxels model with double voids. *(Images are created by the authors using Solidworks Educational Version 30.3.1.2 2022-23).*

With the same overall dimensions, the low bound voxel provides the highest stiffness and the lowest deflection, and for all voxelization settings, the bean can be considered as the same solid structure as depicted in Fig. 5a. Similarly, the high bound provides the lowest stiffness and the highest deflection. Therefore, the beam structure with the largest hollow geometry for the cells is considered as the high bound. The high bound for the symmetric and asymmetric single void 20 voxel model is shown in Fig. 5b, and the high bound for the double void 20 voxel model is shown in Fig. 5c. Due to the increased voxel numbers, the side layer structures of each voxel provide more internal support, which leads to higher stiffness and smaller deflection. The high bound for 40 voxels settings are shown in Fig. 5d,e.

### Material properties

The standard fused deposition modelling material option based on polylactic acid (PLA) is selected as the test material. The material property values are set to: Young's modulus = 3600 MPa, Poisson's ratio = 0.35, and density = 1240 kg/m^3^ ^56^. This choice is based on the fact that it is easy to demonstrate the physical printing of the structures and experimental validation of the analytical and numerical predictions.

### Experimental conditions and initial and target sets

**Figure 6:**
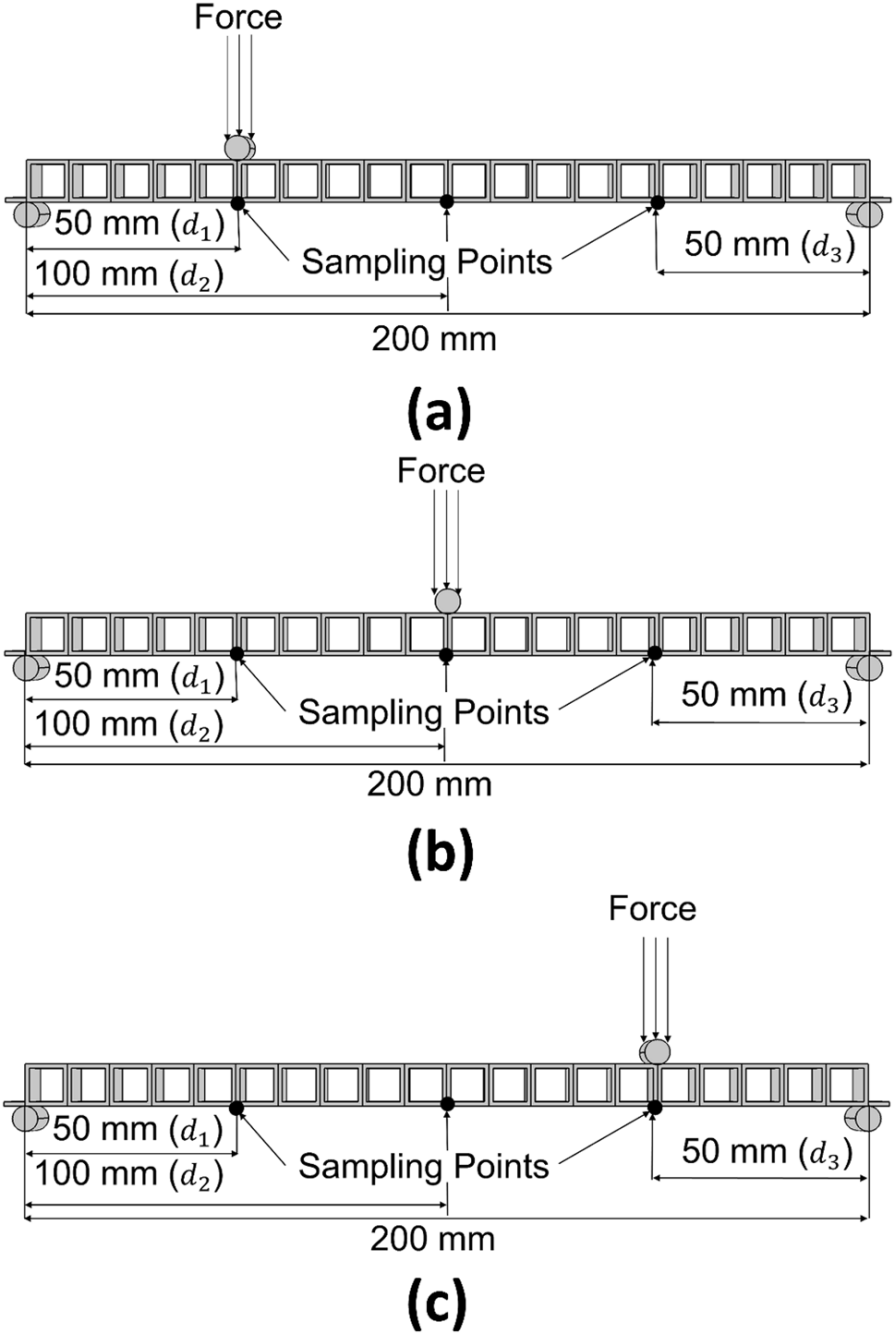
The 20 voxel beam shows the three load and deflection sampling points at the 1/4th, 1/2, and 3/4th distances along the length of the beam structure.

As illustrated in Fig. 6, the beam bending behaviour is evaluated in a standard three-point bending test scenario. Three sampling points are considered located at the 1/4, 1/2, and 3/4 distances from either ends along the length of the beam structure to test the efficiency of the proposed structural optimisation method to control the deflection behaviour of the entire beam. The beam deflection response is controlled during the structural optimisation to provide the exact desired deflections at three selected points with the applied load at three different locations, as shown in Fig. 6 for the three-point bending cases simulated in COMSOL Multiphysics. The load of 100 N distributed over an area is applied to the beam through a roller with a diameter of 6 mm. As shown in Fig. 6, this load is applied at the 1/4th (load case 1 as shown in Fig. 6a), 1/2 (load case 2 as shown in Fig. 6b), and 3/4th (load case 3 as shown in Fig. 6c) distances along the length of the beam simply supported on rollers at both ends. The beam bottom plate is slightly extended, providing extra flanges at the ends for additional support, which has been implemented in both the numerical simulations and experimental tests involving laser sintered polymer prototypes. Three load locations are used and deflections at three points are used with the loading case to compare the experimental and numerical responses. This results in a total of 9 dflection responses compared between preset, numerical, and experimental values. Consequently, the deflections at three specific points under three loading conditions result in a challenging multi-dimensional and multi-objective optimisation problem. Notably, the multiple load cases are designed to test the capability of the proposed structural optimisation method to converge on a single structured beam responding with the desired deflection behaviour under multiple loading conditions.

The CMA-ES evolutionary process starts from an initial configuration, where the height of the hollow structure of each voxel ($H_{h}$) in the initial population is randomly generated, ranging from a full solid voxel to the one with a hollow structure within the boundary limits explained previously. In accordance with these limits, the parametric values of the voxel geometries are constrained to the values listed in Table 2 during the evolutionary search. The voxelised beam structures corresponding to these high and low bound limits are illustrated in Fig. 5. The resulting ranges of deflection in multiple load cases and voxel geometry settings, as calculated by the finite element analysis, are listed in Table 3. The target sets (TS) tested for the 20 voxel single void structure model are listed in Table 4. The initial target sets for the desired beam deflection values are selected as the midpoints (TS1) between the low and high bound limit results and then varied around the midpoint. In TS2 and TS3, The desired beam deflection target sets are varied at + 0.2 mm and − 0.2 mm from the midpoint, respectively. In TS4 and TS5, The desired beam deflection target sets are varied at + 0.4 mm and − 0.4 mm from the midpoint, respectively. For the 40 voxel and double void structured models, the high bound limit is different. Therefore, different target sets are used to verify and compare the performance, and the final target sets used for 40 voxel models are listed in Table 5. Similar to the single void structured model, TS6 is the midpoint between the results of the low and high bound limits. The target set for desired beam deflection is varied at + 0.2 mm in TS7, which is the largest possible variation for the double void model.

**Table 2:** The limits of the voxel geometric parameters for the beam models.

Geometric parameter (mm)	20 voxel			40 voxel		
	High bound (single symmetric and asymmetric void)	High bound (double void)	Low bound	High bound (single symmetric and asymmetric void)	High bound (double void)	Low bound
Void length $L_{v}$	10	10	10	5	5	5
Voxel width $W_{b}$	30	30	30	30	30	30
Voxel Height $H_{b}$	10	10	10	10	10	10
Flange thickness $T_{b}$	1	1	N/A	1	1	N/A
Side thickness $T_{s}$	1	1	N/A	1	1	N/A
Voxel height $H_{v}$	8	3.5	N/A	8	3.5	N/A
Middle layer thickness $T_{m}$	N/A	1	N/A	N/A	1	N/A

The guidance on the meaning of the parameters refers to Fig. 4.

**Table 3:** The limiting ranges of beam deflections at the sampling points resulting from the voxel geometrical parameters calculated via FEM simulation by COMSOL Multiphysics as listed in Table 2.

Unit: mm	20 voxels	20voxels	40 voxels	40 voxels	Low bound (All setup)
	Single void settings	Double void settings	Single void settings	Double void settings	
	High bound	High bound	High bound	High bound	
Loading 1 Sampling point 1 (L1S1)	− 2.474	− 1.807	− 1.437	− 1.151	− 0.469
Loading 1 Sampling point 2 (L1S2)	− 2.241	− 1.714	− 1.515	− 1.241	− 0.514
Loading 1 Sampling point 3 (L1S3)	− 1.172	− 0.875	− 0.809	− 0.634	− 0.242
Loading 2 Sampling point 1 (L2S1)	− 2.235	− 1.708	− 1.509	− 1.235	− 0.511
Loading 2 Sampling point 2 (L2S2)	− 3.812	− 2.888	− 2.421	− 1.987	− 0.831
Loading 2 Sampling point 3 (L2S3)	− 2.235	− 1.708	− 1.509	− 1.235	− 0.511
Loading 3 Sampling point 1 (L3S1)	− 1.172	− 0.875	− 0.809	− 0.634	− 0.242
Loading 3 Sampling point 2 (L3S2)	− 2.241	− 1.714	− 1.515	− 1.241	− 0.514
Loading 3 Sampling point 3 (L3S3)	− 2.474	− 1.807	− 1.437	− 1.151	− 0.469

**Table 4:** The target deflection sets for 20 voxel single void structured beam.

Unit (mm)	L1S1	L1S2	L1S3	L2S1	L2S2	L2S3	L3S1	L3S2	L3S3
Low bound	− 0.469	− 0.514	− 0.242	− 0.511	− 0.831	− 0.511	− 0.242	− 0.514	− 0.469
High bound	− 2.474	− 2.241	− 1.172	− 2.235	− 3.812	− 2.235	− 1.172	− 2.241	− 2.474
Midpoint (TS1)	− 1.471	− 1.377	− 0.707	− 1.373	− 2.321	− 1.373	− 0.707	− 1.377	− 1.471
Midpoint + 0.2(TS2)	− 1.271	− 1.177	− 0.507	− 1.173	− 2.121	− 1.173	− 0.507	− 1.177	− 1.271
Midpoint − 0.2(TS3)	− 1.671	− 1.577	− 0.907	− 1.573	− 2.521	− 1.573	− 0.907	− 1.577	− 1.671
Midpoint + 0.4(TS4)	− 1.071	− 0.977	− 0.307	− 0.973	− 1.921	− 0.973	− 0.307	− 0.977	− 1.072
Midpoint − 0.4(TS5)	− 1.871	− 1.777	− 1.107	− 1.773	− 2.721	− 1.773	− 1.107	− 1.777	− 1.871

**Table 5:** The target deflection sets for 40 voxel beam.

Unit (mm)	L1S1	L1S2	L1S3	L2S1	L2S2	L2S3	L3S1	L3S2	L3S3
Low bound	− 0.469	− 0.514	− 0.242	− 0.511	− 0.831	− 0.511	− 0.2420	− 0.514	− 0.469
High bound	− 1.437	− 1.515	− 0.809	− 1.509	− 2.421	− 1.509	− 0.809	− 1.515	− 1.437
Midpoint(TS6)	− 0.953	− 1.015	− 0.526	− 1.01	− 1.626	− 1.01	− 0.526	− 1.015	− 0.953
Midpoint + 0.2 (TS7)	− 0.753	− 0.815	− 0.326	− 0.81	− 1.426	− 0.81	− 0.326	− 0.815	− 0.753

## Results and discussion

### Tuning the algorithm

The standard CMA-ES algorithm is used in this work. The step size control and covariance update parameters are set as default values and will update in each iteration based on the fitness value obtained. The optimization performance is highly dependent on the population size and iteration number, which govern the range of the search domain. The voxel number is also a vital parameter, which is proportional to the number of degrees of freedom. However, large population size, iteration number, and voxel number can lead to excessive computational times. It may be noted that the abbreviations TS1 to TS7 are used in the following discussions for brevity and they refer to the respective deflection sets identified and listed in Tables 3 and 4.

**Figure 7:**
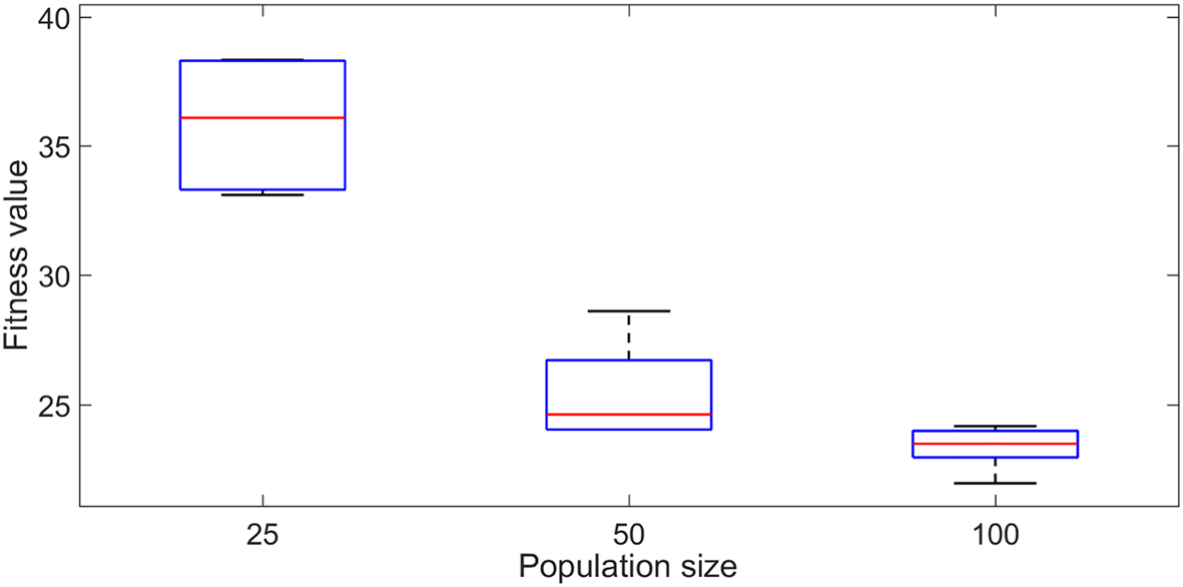
The effect of population size on the fitness value for TS4 using the 20 voxel single symmetric model. The solid lines in the middle indicate the median fitness value, and the boxes represent the 25th and 75th percentiles.

A small number of initial numerical experiments were conducted to identify the influences of each of the optimisation scheme parameters. The 20 voxel model with a single symmetric voxel setting and the target set TS4 are used in the initial tests for identifying the most efficient population size for the optimisation. TS4 contains the highest variance between each sampling point, which is the most challenging target set for the optimisation scheme. The results of five independent trials are summarized in Fig. 7, where the lower the fitness value, the better the results. It is clearly evident that when the population size is doubled to 50, the optimisation method can provide a closer match and at an approximately 50% lower fitness value compared with the trials with a population size of 25. With a further increase in the population size to 100, both the variance and fitness value further decreased by approximately around 10%. However, with increasing population size, the computational time also increases proportionately, leading to heavy computational constraints. Based on these observations, the population size is fixed at 50 in all the rest of the tests presented next in order to avoid excessive computational times and associated time constraints.

**Figure 8:**
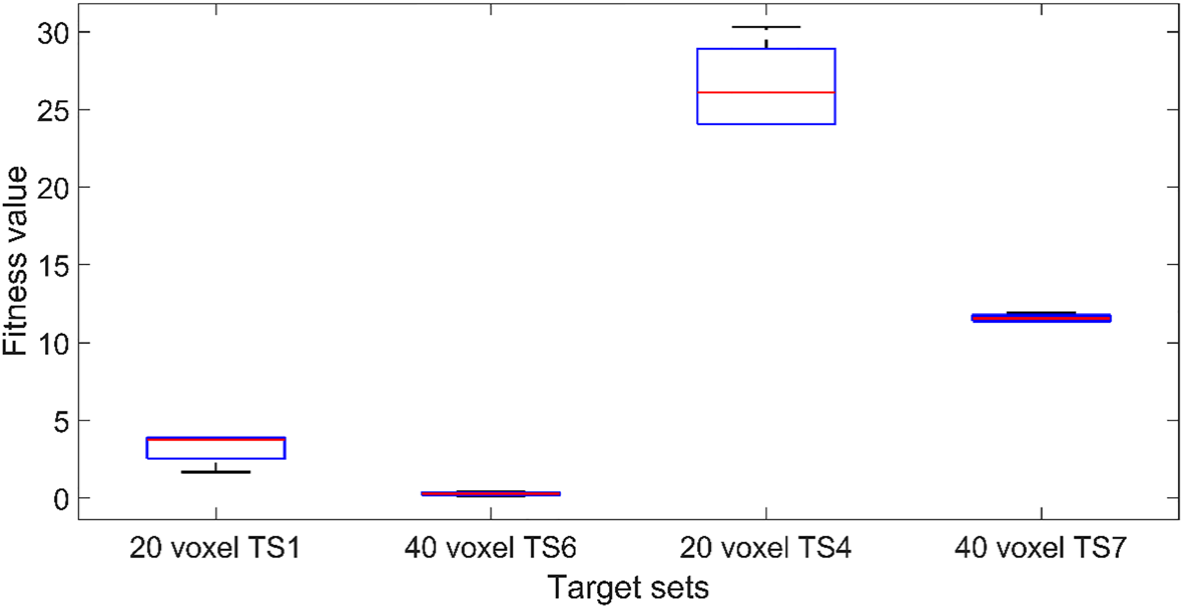
The effects of voxel number on the fitness value. The solid lines in the middle indicate the median fitness value, and the boxes represent the 25th and 75th percentiles.

**Figure 9:**
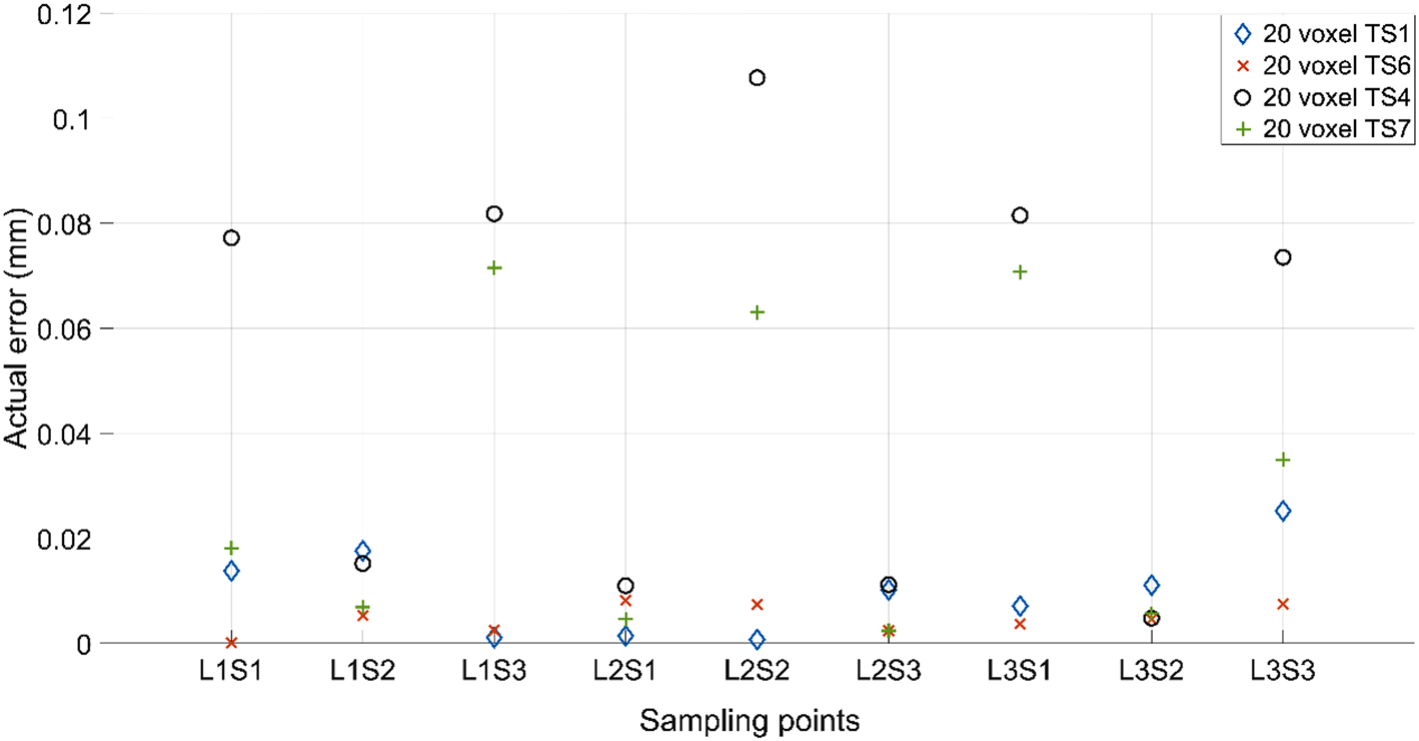
The mean of the absolute difference between the deflection from numerical results and the corresponding target deflection in the nine sampling points.

In terms of the voxel numbers, both the 20 voxel and 40 voxel models are tested. The midpoint (TS1 and TS6) and the target set with the highest achievable variance from the midpoint (TS4 and TS7) are selected, respectively, for the 20 and 40 voxel models. The results of five independent trials are illustrated in Fig. 8. Once again, based on the premise that the lower the fitness value, the better, it is clear that the 40 voxel model can provide a superior result compared to the 20 voxel model in both variance and fitness values. However, as depicted in Fig. 9, the mean of the difference between the actual deflection and the corresponding target deflection at the nine sampling points is less than 0.1 mm in most cases. It indicates that both 20 voxel and 40 voxel models can provide satisfactory results in matching the target deflection sets. Computationally, the 40 voxel model is significantly more expensive compared to the 20 voxel model in terms of both time and other constraints. The computation time of the fitness function for a single evaluation is increased from 9 to 33 s on a standard Intel i7 9700 k processor when moving from 20 to 40 voxel cases. As a balance between the computational costs and the performance of the optimisation scheme, the 20 voxel model is used with a population size of 50 as the standard setting in the CMA-ES optimisation in all the numerical experiments following.

### Beam with standard single symmetric void voxels

**Figure 10:**
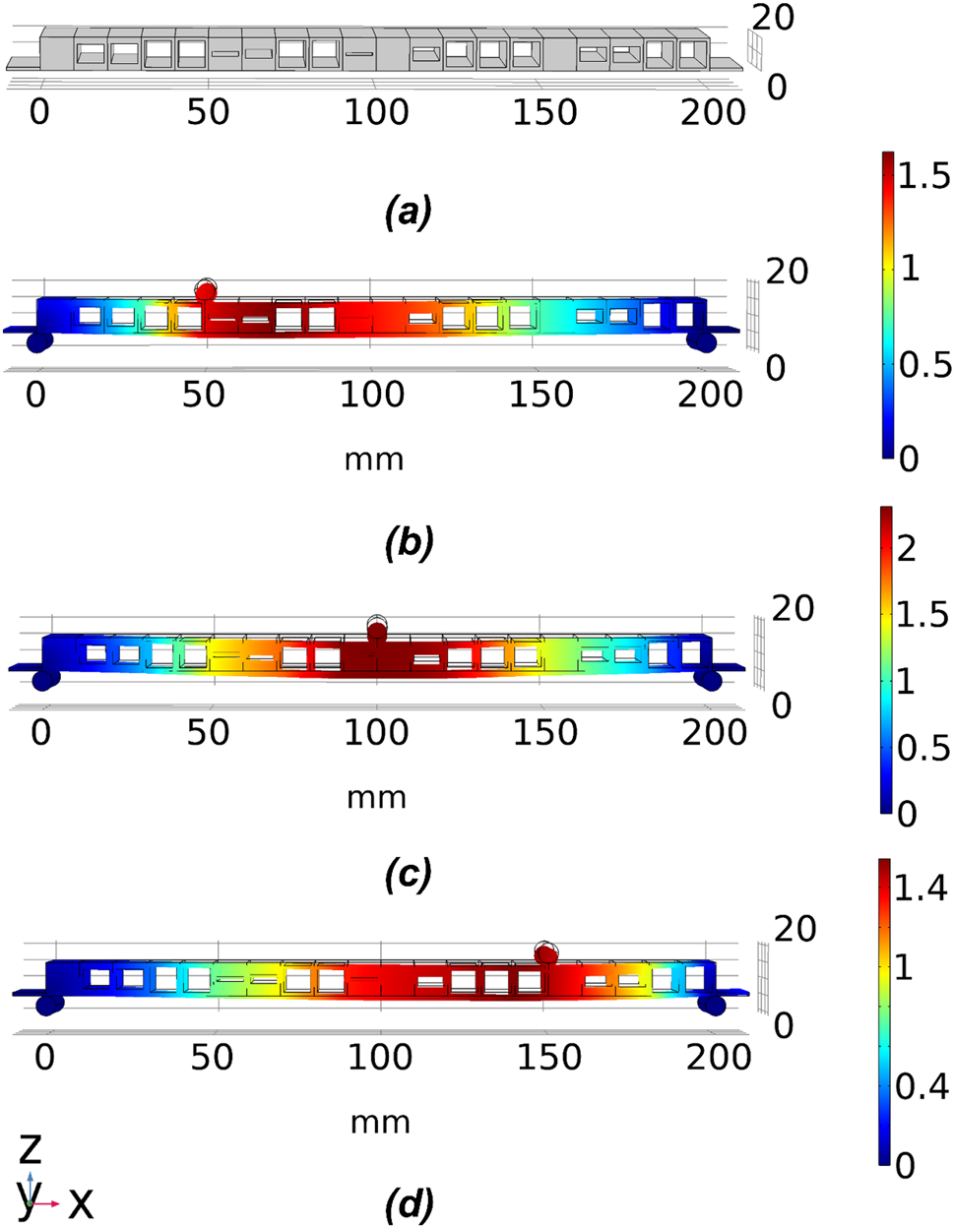
The optimised beam structure for TS1. (**a**) the geometry of the optimised beam model and (**b**–**d**) deflection patterns under loading conditions 1–3.

**Figure 11:**
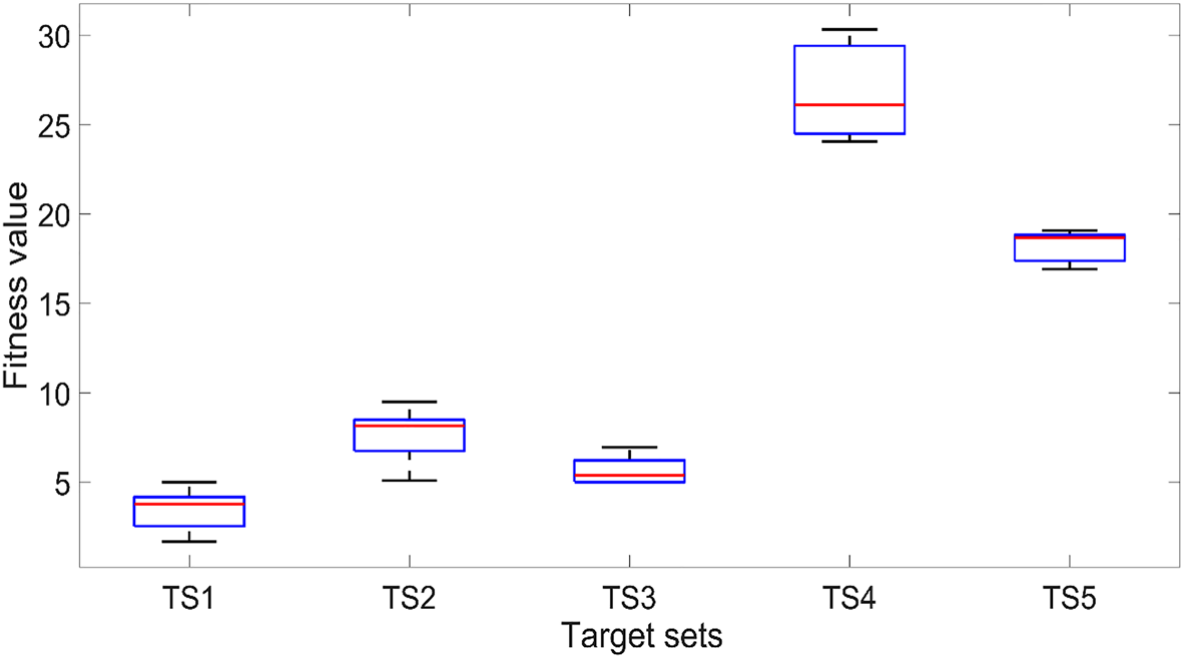
Comparison of fitness values with the 20 voxel single symmetric void model for target deflection sets TS 1 to TS5. Each box plot represents the results of 5 trials for each target set. The lower the fitness value, the closer the obtained deflection sets to the target deflection sets. The solid lines in the middle indicate the median fitness values, the top and bottom of the boxes represent the 25th and 75th percentiles.

**Figure 12:**
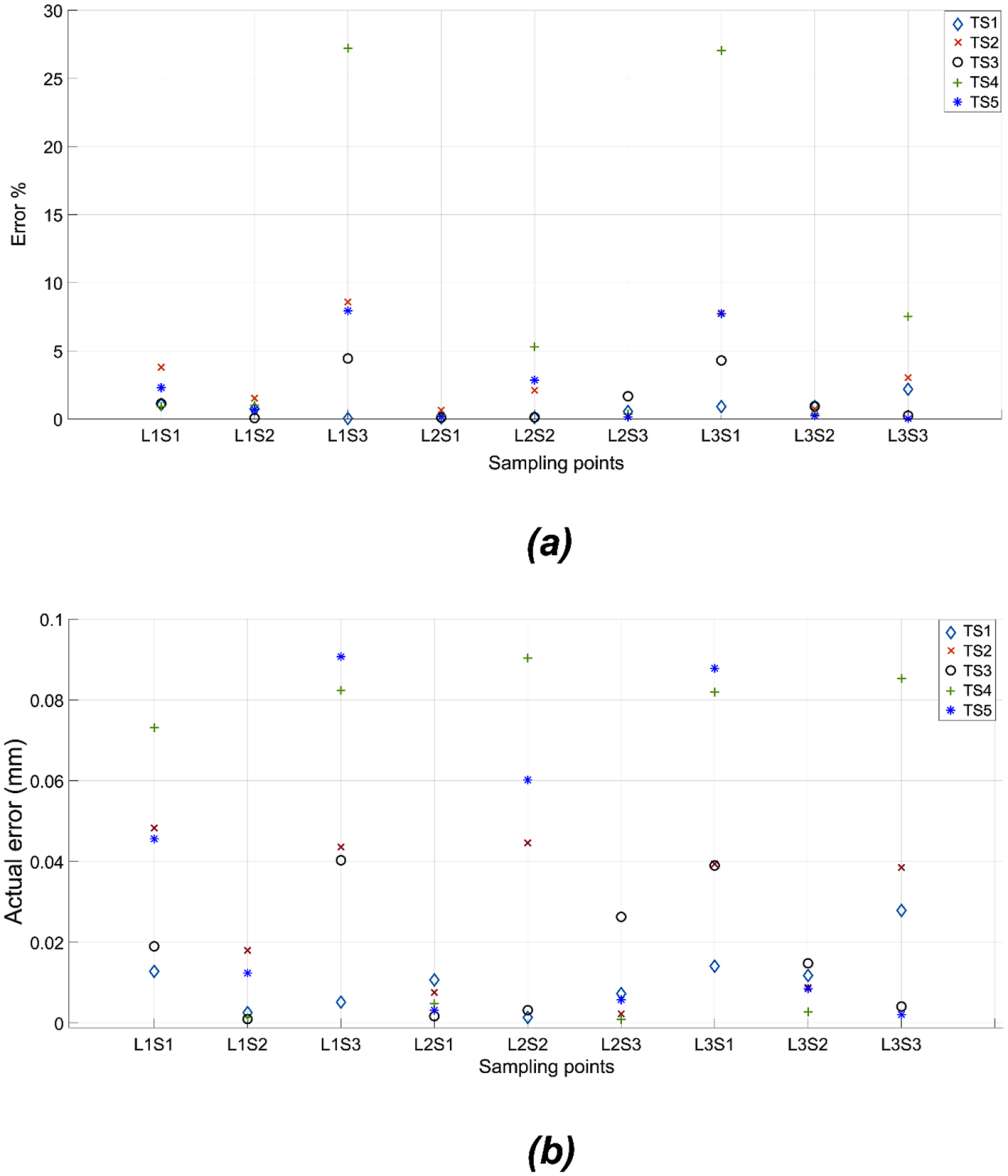
The mean of the difference between the deflection in nine sampling points and the corresponding target deflections for TS1 to TS5 in 20 voxel symmetric single void models is expressed as (**a**) percentage and (**b**) actual difference in deflections. Each data point corresponds to the mean value of 5 independent trials.

The deflection target sets TS1 to TS5 are used for the numerical optimisation based on the standard single symmetric void beam model. The structural form of the optimised beam for TS1 is illustrated in Fig. 10a and the deflection patterns with the three loading cases in Fig. 10b–d. The fitness values obtained with the different target sets as compiled in Fig. 11 indicate that the result for TS1 attained the lowest fitness value. The target sets TS4 and TS5 ended up with the highest fitness values, indicating the highest error levels. Referring back to the listings of the deflection ranges in Table 4, TS4 and TS5 have the largest variation from the midpoint between the low and high bound limits and the deflection targets with sampling points L1S3 and L3S1 for TS4 and TS5 are close to the low and high bound limits respectively, while the deflections at the other sampling points still remain relatively close to the midpoint. For example, to match with the target deflections corresponding to the settings of L1S3 and L3S1 cases in TS4, the optimisation scheme needs to create the largest void dimensions for obtaining the lowest overall stiffness of the structure while the stiffness matrix of the beam structure also has to maintain higher stiffness for matching the target deflection at other sampling points. This conflict between the demanding conditions at the opposite ends at different sampling points is the main reason for the higher error in the trials TS4 and TS5. It is pertinent to point out that the sampling points L1S3 and L3S1 in TS4 and TS5 are the major sources of error in Fig. 12a, adding strength to the above arguments. The optimised beam provided the deflections to closely match the preset conditions, with an error of less than 10% in all other sampling points and a much lower error in many cases.

TS4 and TS5 both have the same variation (0.4 mm) from the midpoint between the low and high bound. However, as shown in Fig. 12a, it can be seen that the proposed optimisation scheme obtained better performance in TS5. The main reason is that TS4 has a smaller deflection value compared with TS5, which represents a higher error in percentage. From Fig. 12b, it can be seen that the optimised beam obtained a similar error for matching the conditions of TS4 and TS5. Overall, as shown in Fig. 12b, the proposed optimisation scheme provided satisfactory accuracy levels in matching the deflection profiles with any case of the target sets within a maximum deviation of 0.1 mm.

### Beam with double and asymmetric void voxels

**Figure 13:**
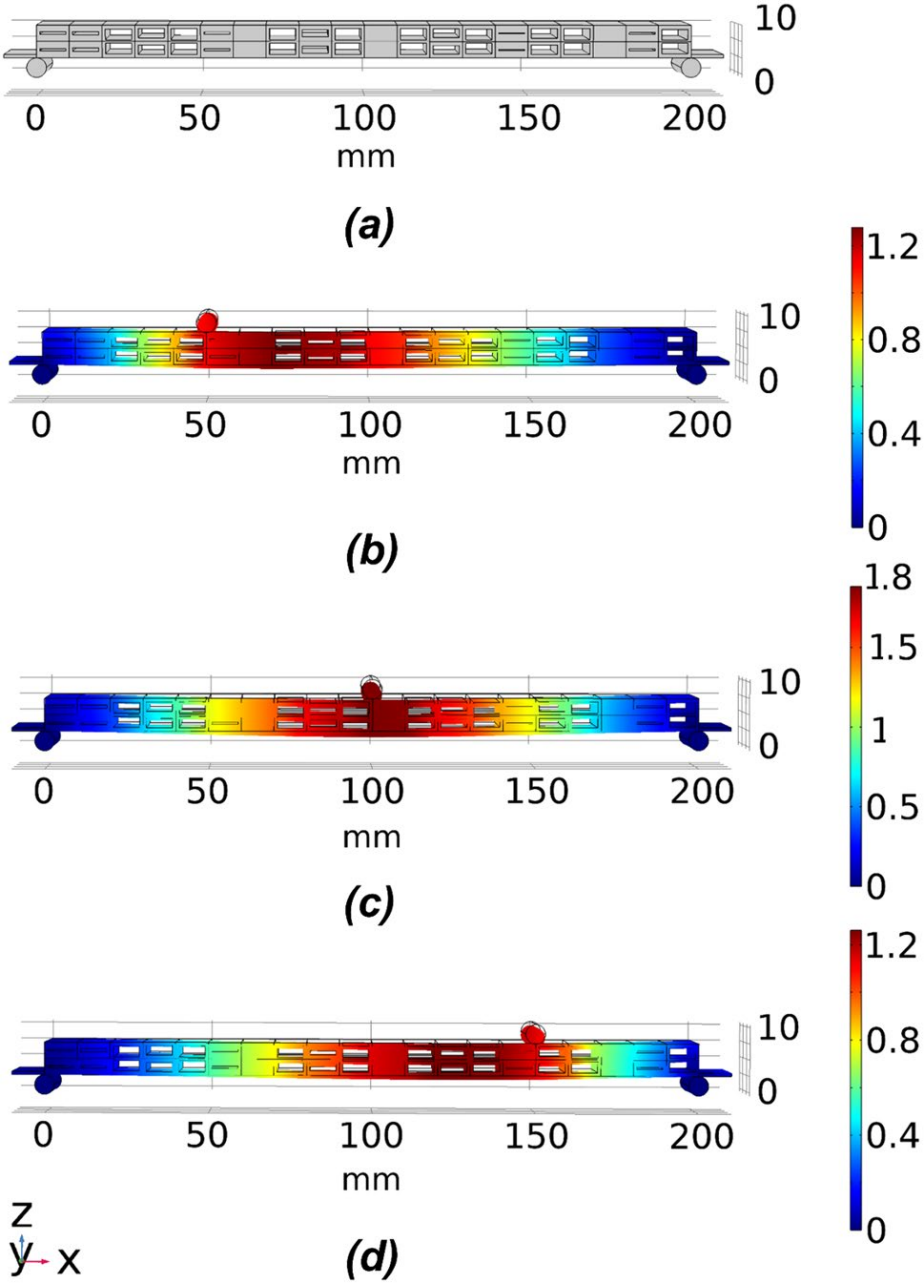
An example of the optimised 20 voxel double void beam model for the target set TS8. (**a**) The geometry of the optimised beam model and (**b–d**) are deflection plots under loading conditions 1 to 3, respectively.

For the double void model, the thickness of the middle layer $T_{m}$ serves as an extra parameter in the optimisation scheme, which provides an additional degree of freedom and better control of the design domain while also adding more complexity and a further dimension in the search space of the proposed optimisation scheme. As a result of the extra middle layer in the cellular structure, the high bound limit of the achievable deflection is different. As listed in Table 6, different target sets, TS8 and TS9, are used. TS8 corresponds to the midpoint between the low and high bound limits of the achievable deflections, while TS9 is the target set with + 0.2 mm variation from the midpoint, which is the highest achievable variation for the double void voxel model. An example of the optimised 20 voxel double void beam structure is depicted in Fig. 13. The resulting beam structure is made up of double void voxels with varying internal dimensions for the voids that are symmetric about the central web.

**Table 6:** The target deflection sets for double void 20 voxel beam.

Unit (mm)	L1S1	L1S2	L1S3	L2S1	L2S2	L2S3	L3S1	L3S2	L3S3
Low bound	− 0.469	− 0.514	− 0.242	− 0.511	− 0.831	− 0.511	− 0.2420	− 0.514	− 0.469
High bound	− 1.807	− 1.714	− 0.875	− 1.708	− 2.888	− 1.708	− 0.875	− 1.714	− 1.807
Midpoint (TS8)	− 1.138	− 1.114	− 0.559	− 1.109	− 1.859	− 1.109	− 0.559	− 1.114	− 1.138
Midpoint + 0.2 (TS9)	− 1.338	− 1.314	− 0.759	− 1.309	− 2.059	− 1.3095	− 0.7585	− 1.314	− 1.338

**Figure 14:**
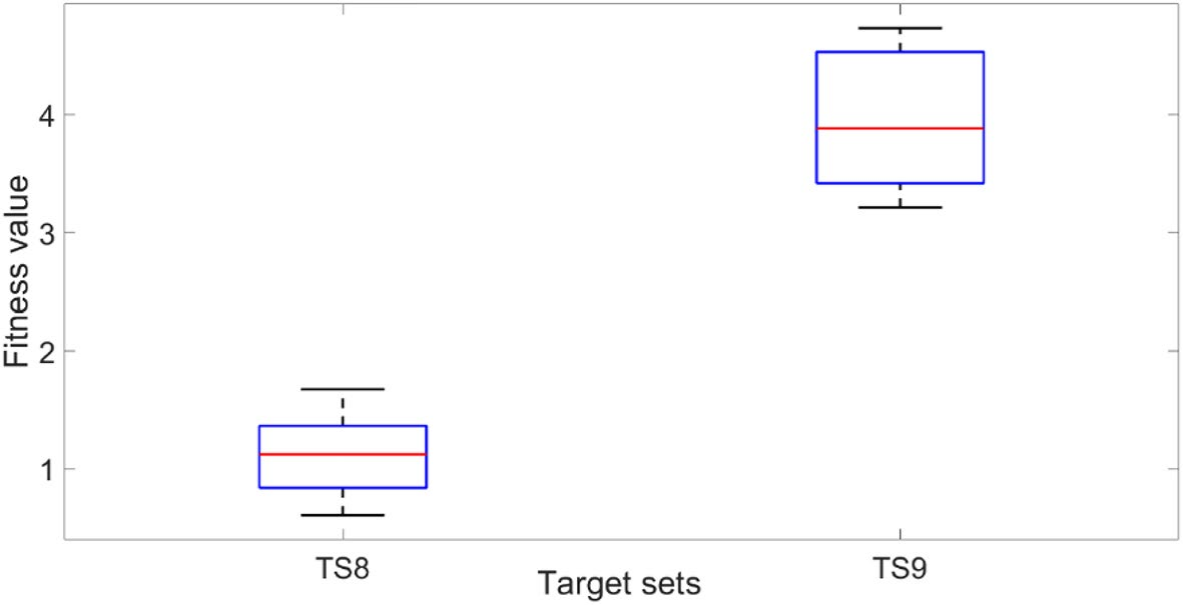
Comparison of fitness values for 20 voxel double void model for target deflection sets TS8 and TS9. Each box plot represents the results of 5 trials for each target set. The lower the fitness value, the closer the obtained deflection sets were to the target deflection set. The solid lines in the middle indicate the median fitness values, the top and bottom of the boxes represent the 25th and 75th percentiles.

**Figure 15:**
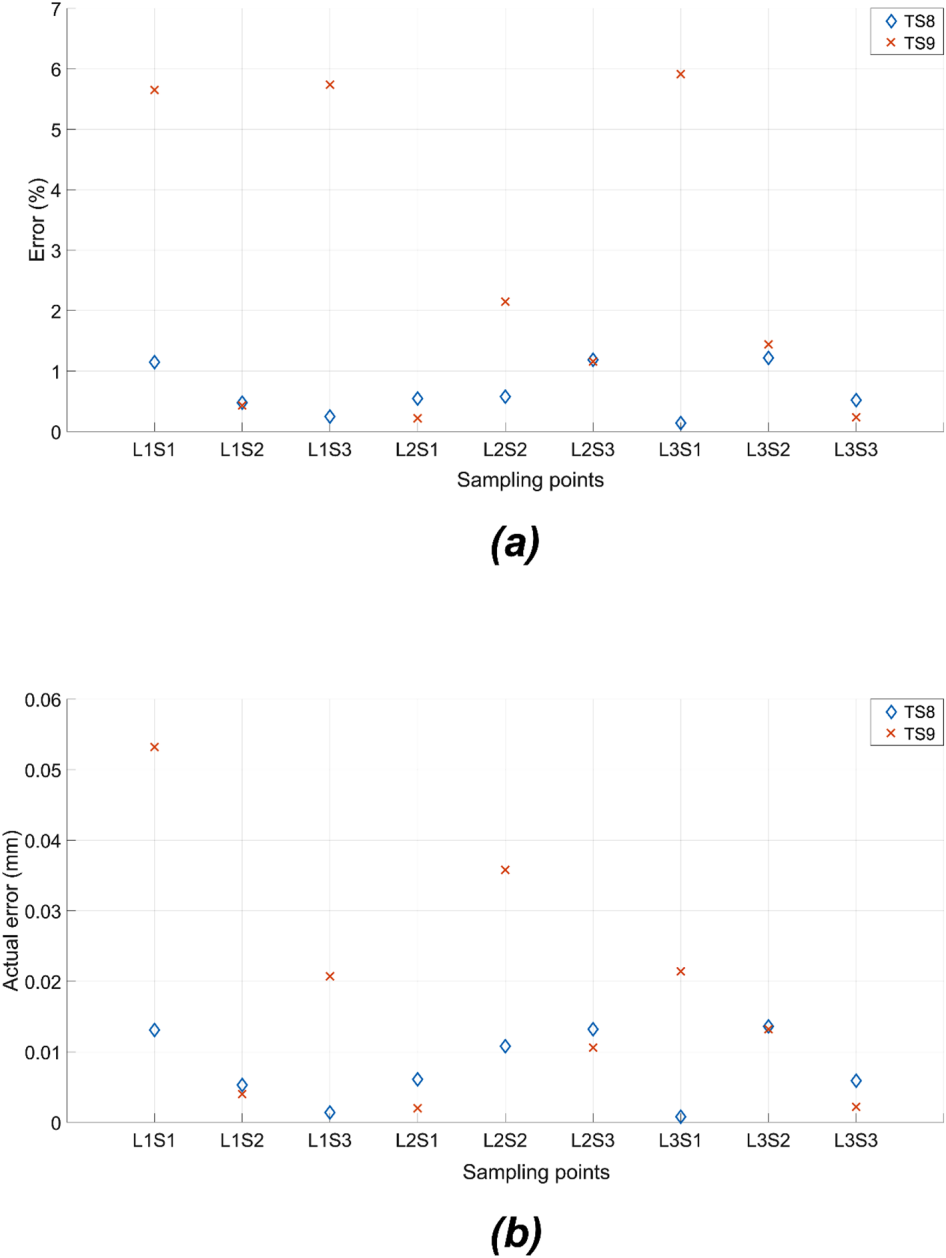
The mean of the difference between the deflection in nine sampling points and the corresponding target deflections for TS8 and TS9 in 20 voxel double void models is expressed as (**a**) percentage and (**b**) actual deflection difference. Each data point corresponds to the mean value of 5 independent trials.

As shown in Fig. 14, the fitness values for target sets TS8 and TS9 are approximately 1 and 4, respectively. Compared with the single symmetric void model, the double void voxel model obtained better performance with the same number of voxels. Evidently, with the additional manipulation of the thickness of the middle layer in the double void cellular structure, the optimisation scheme had better control over the overall stiffness matrix, which in turn governs the deflection profile of the beam. The percentage and actual deviations between the target and achieved values resulting from the FE simulations of the optimised beam structures are presented in Fig. 15a,b, respectively. The results in Fig. 15a,b clearly indicate the better performance of the CMA-ES optimisation scheme in converging on the optimum structural geometry of the cellular beam, effectively utilising the added degree of freedom from the double void geometry. The optimised cellular beam structures achieved deflection patterns close to the values of the desired deflection target sets TS8 and TS9. At all the sampling points, the percentage and actual deviations between the predicted and desired deflection levels (errors) are less than 6% and 0.06 mm, respectively.

**Figure 16:**
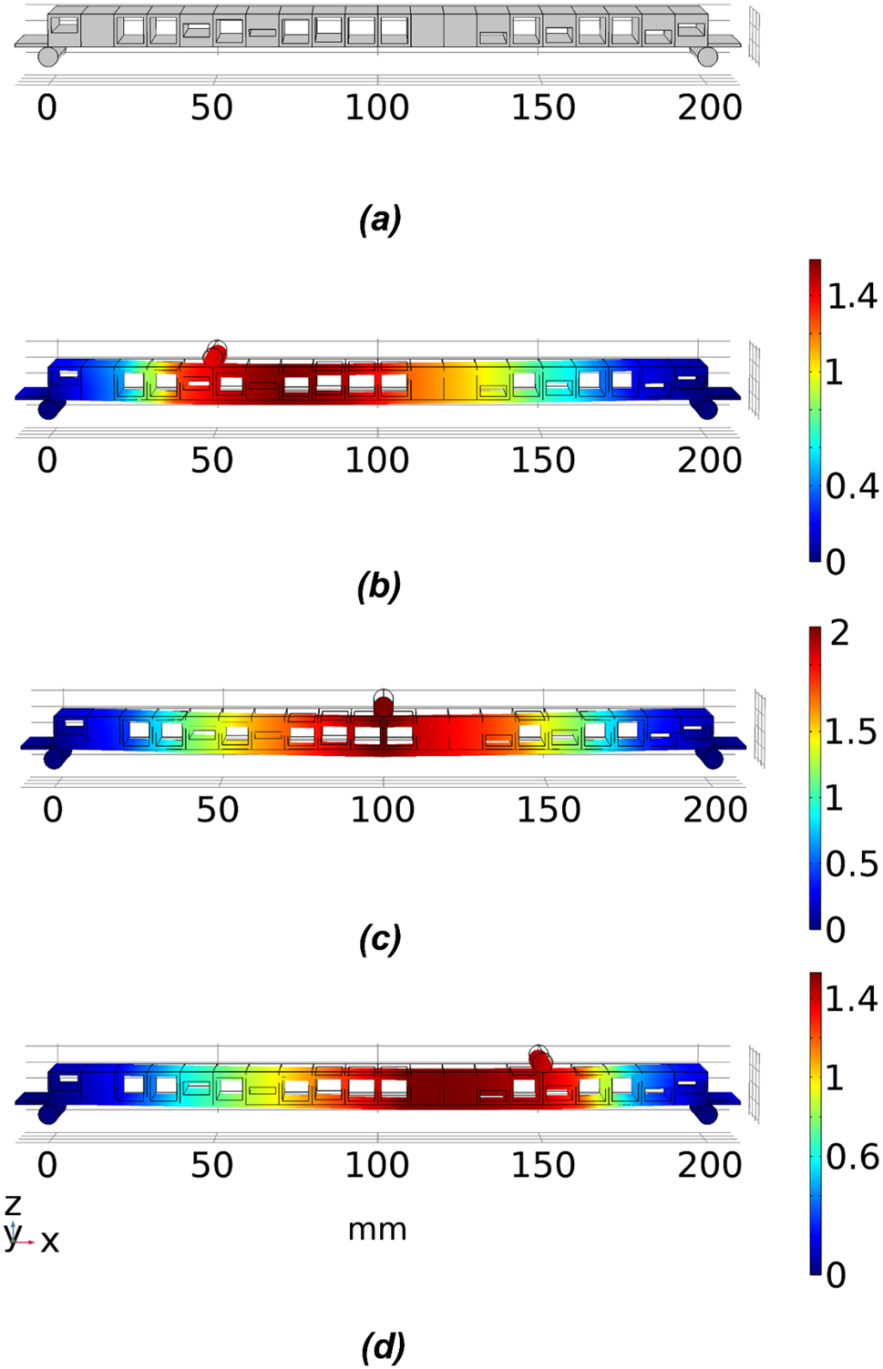
An example of the optimised 20 voxels asymmetric single void beam model for target set TS1. (**a**) The geometry of the optimised beam model, and (**b**–**d**) is the deflection under loading conditions 1–3.

For the asymmetric single void model, referring back to Fig. 4 again, the thickness of the bottom web, $T_{b}$ can be used as an additional parameter to control the location of the void within the voxel, which will double the geometric parameters for optimisation compared to the single void symmetric cellular structure. This additional parameter $T_{b}$ is again expected to allow the optimisation scheme to gain better control over the deflection behaviour of the beam model while also increasing the complexity and the dimensions of the searching problem. The asymmetric single void model shares the same low and high bound limits for the deflections as the symmetric single void voxel model. Therefore, the same target deflection sets TS1 and TS4 as employed in “Experimental conditions and initial and target sets” section are used. An example of the optimised cellular geometry of the beam with the asymmetric single void structure is depicted in Fig. 16a and the deflection profiles in Fig. 16b–d as obtained with the three loading cases. The variation of the geometry of the cells based on asymmetric positioning of the voids as obtained by the optimisation scheme is clearly reflected in Fig. 16a.

**Figure 17:**
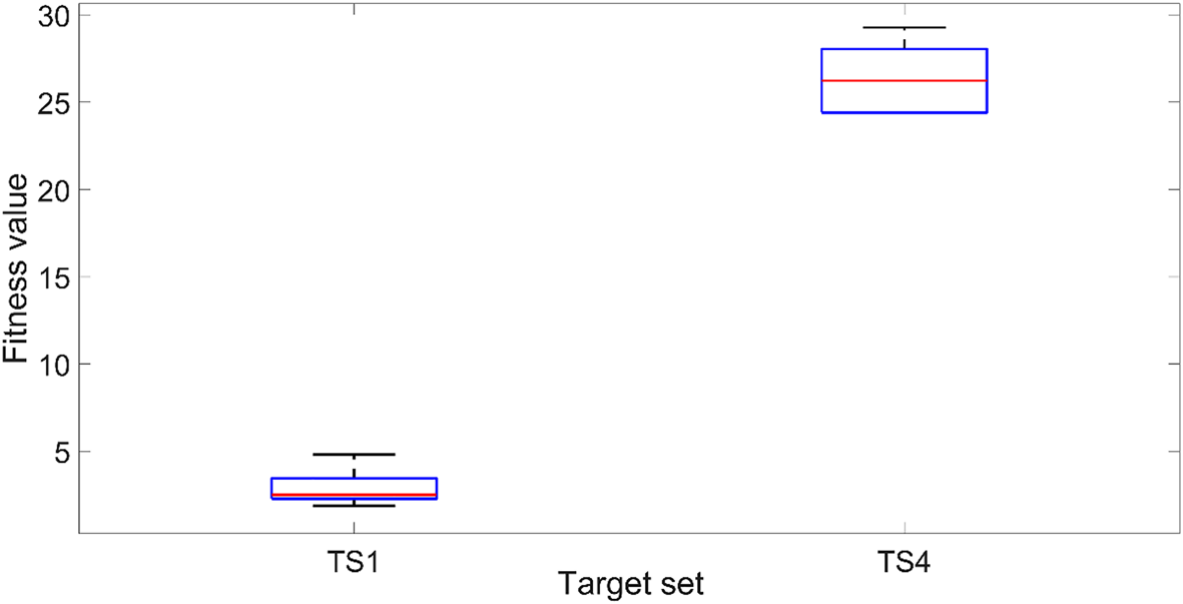
Comparison of fitness values for 20 voxel asymmetric single void beam model for target deflection sets TS1 and TS4. Each box plot represents the results of 5 trials for each target set. The lower the fitness value, the closer the obtained deflection sets were to the target deflection set. The solid lines in the middle indicate the median fitness values, the top and bottom of the boxes represent the 25th and 75th percentiles.

**Figure 18:**
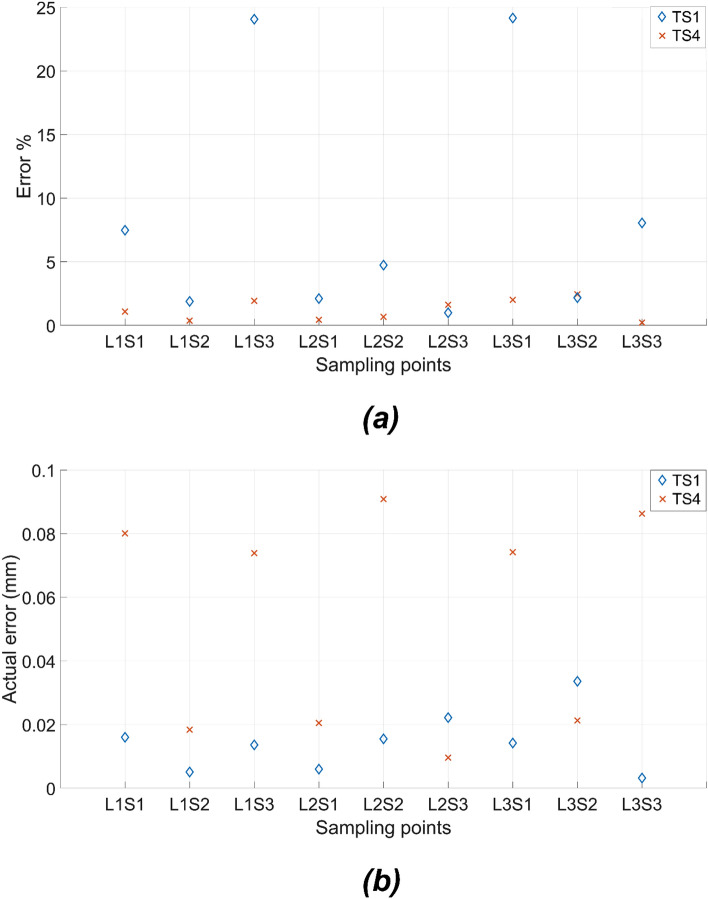
The mean of the differences between the deflection at nine sampling points and the corresponding target deflections for TS1 and TS4 in 20 voxel asymmetric single void beam model is expressed as (**a**) percentage and (**b**) actual difference in the deflection. Each data point corresponds to the mean value of 5 independent trials.

As illustrated in Figs. 17 and 18, the optimisation with the asymmetric single void model obtained similar trends in results as the standard symmetric single void model. The maximum deviation between predicted and target results is less than 10% or 0.08 mm in most of the cases. Though the optimisation scheme was effective in handling the additional degrees of freedom, there is no specific improvement in the convergent results based on the asymmetric model compared to the symmetric single void model. The main reason for this is the fact that there is no substantial variation in the property ranges that can actually be obtained by shifting from the symmetric to the asymmetric variations in the single void cellular geometries.

The results for sampling points L1S3 and L3S1 still suffered from larger errors. Similar to the results based on the standard symmetric single void model, the targets for sampling points L1S3 and L3S1 are close to the high bound deflection limits, while the targets for the other sampling points still remain relatively close to the midpoint between the low and high bound limits. To match with the target values of the sampling points L1S3 and L3S1, the optimisation scheme is required to use more void structures that provide higher deflection, while fewer void structures for lower deflection are required in nearby voxels to match the targets in other sampling points. Evidently, even with the additional control over the beam structure, the conflicts between the target deflection profiles of different sampling points play significant roles in the performance of the optimisation scheme.

### Additive manufacturing and experimental validation

Considering the time and cost constraints, the physical reproduction of the cellular beam structures by additive manufacturing and the experimental validation of deflection profiles based on three-point bending tests are confined to the standard symmetric single void beam prototypes of 20 voxels. All the beam test pieces are printed with a build orientation in which no external support structures are needed, using the commercial PLA filaments based on a CreatBot 3D printer (Model F430) with the critical process parameter settings identified as the best options after a few initial trials and listed in Table 7. The true material properties of the PLA polymer used for the experimental trials are first established by printing a few standard test pieces and conducting the tensile tests. The average Young's modulus thus obtained is widely different from the mechanical properties stated in “Material properties” section earlier and used in all the numerical results discussed in the preceding sections.

**Table 7:** Optimised printing parameters used for the physical prototyping of the 20 voxel beam structures.

Nozzle temperature (°C)	Bed temperature (°C)	Printing speed (mm/s)	Layer Height (mm)	Nozzle diameter (mm)	Infill density (%)
210	60	55	0.2 mm	0.4	100

**Figure 19:**
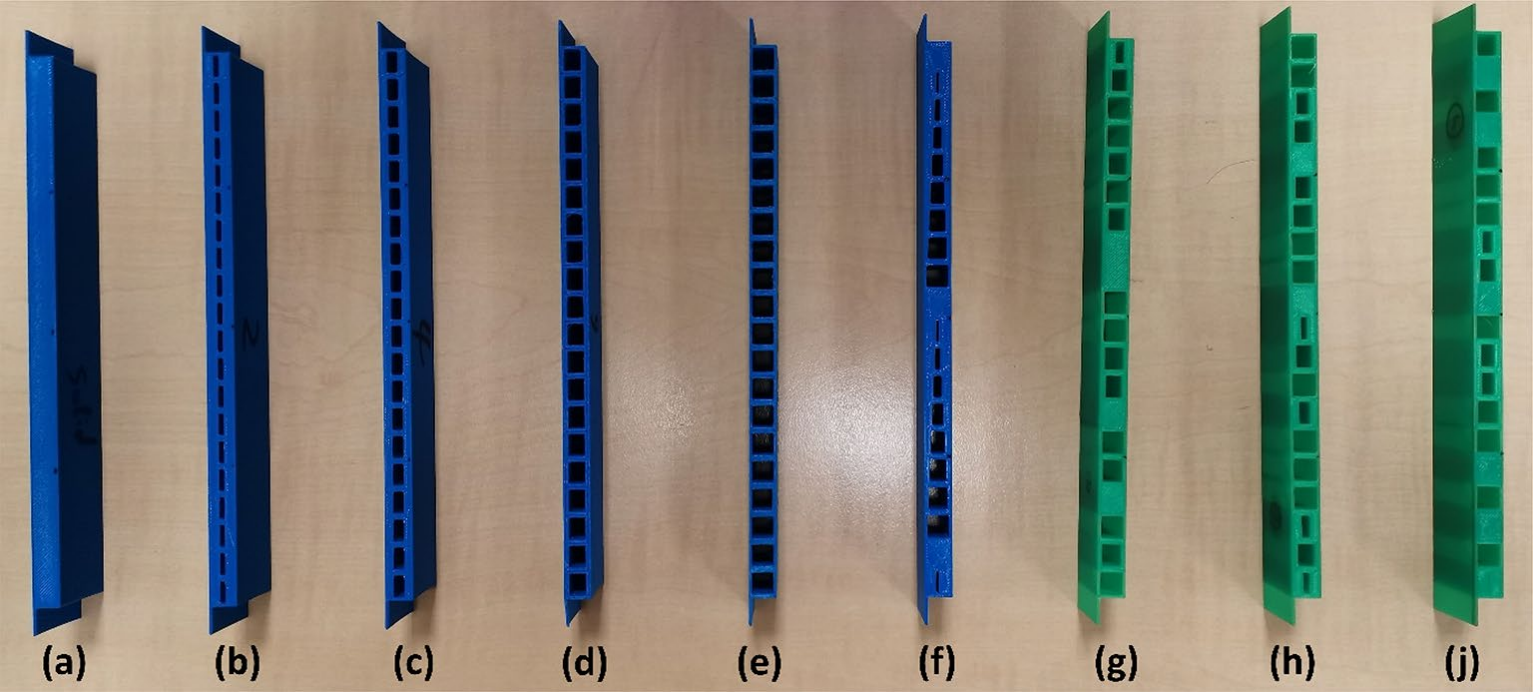
3D printed samples (**a**) low bound 20 voxels single void beam; (**b**–**e**) 20 voxels single void beam with 20% 40% 60% and 80% void respectively; (**f**) 20 voxels single void beam with incremental void sizes; (**g**–**j**) optimised 20 voxels single symmetric void beam for TSE1- TSE3. *(Image is based on the photograph taken by the authors).*

Considering the wide variation in the modulus of elasticity, and also the fact that the varying geometry of the beam has an influence on the effective Young's modulus of the material, a series of tests have to be undertaken. Six beam specimens with solid, 20%, 40%, 60%, and 80% voids, and the incremental void case geometries as depicted in Fig. 19a–f are printed and subjected to three-point bending loads applied at the three experimental points. The deflections at the three locations are recorded for each geometry and load case. Numerical simulations are done with the same six geometries and the three load cases in each, and the best correlation between the experimental and the numerical predictions was obtained by varying the effective Young's modulus value in each case. Eventually, the average Young's modulus at 1600 MPa was found to give the best matching between the experimental and numerical results in all six cases and is adopted as the critical property for the final experimental validation of the beam deflection profiles.

**Figure 20:**
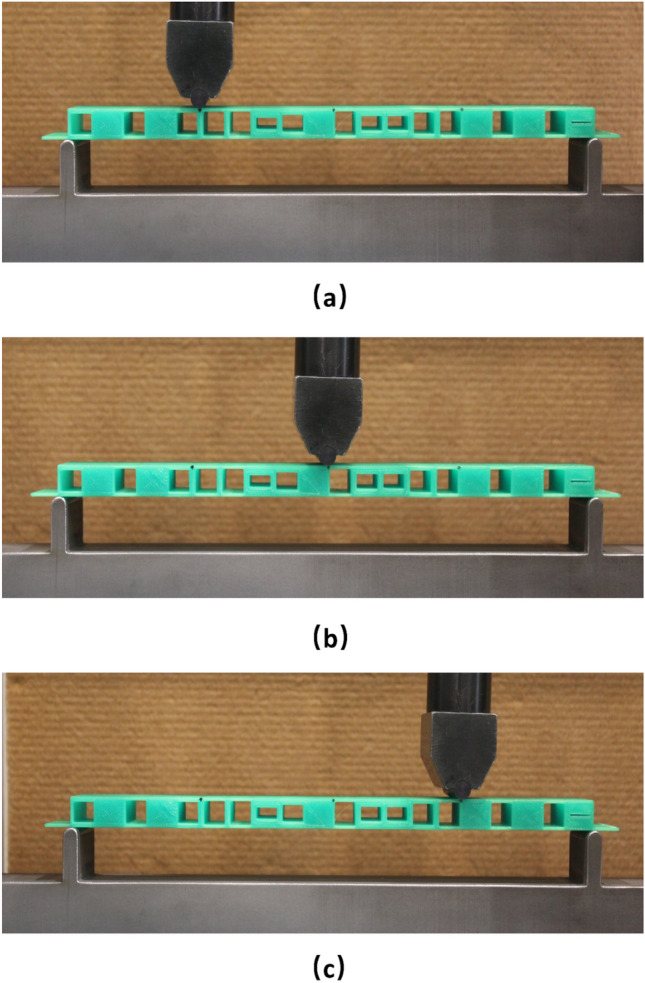
Experimental three-point bending tests; (**a**–**c**) indicate loading points 1 to *3 (Image is based on the photograph taken by the authors).*

Further, the 20 voxel beam case is reevaluated numerically using the newly established material property and the limiting deflection values are reestimated. Based on these newly established low and high bound values, different target deflection values are estimated, as listed in Table 8, for validation through the three-point bending experiments. The target sets at the mid, mid + 0.2 mm and mid-0.2 mm points are referred to as TSE1, TSE2, and TSE3, respectively, to differentiate them from the target sets TS1 to TS3 used earlier. The optimised 20 voxel single symmetric void beams for TSE1 to TSE3 cases based on numerical simulations using these properties and target deflections are physically printed as shown in Fig. 19g–i. The beam loading setups for the three-point bending under loads at different locations are shown in Fig. 20 where each experimental case is repeated three times and the average values are used in the experimental validation that follows.

**Table 8:** The target deflection values for symmetric single void 20 voxel beam reestimated using the true material properties for experimental validation purposes.

Unit (mm)	L1S1	L1S2	L1S3	L2S1	L2S2	L2S3	L3S1	L3S2	L3S3
Low bound	− 1.06	− 1.16	− 0.54	− 1.15	− 1.869	− 1.15	− 0.54	− 1.16	− 1.06
High bound	− 5.57	− 5.04	− 2.64	− 5.03	− 8.58	− 5.03	− 2.64	− 5.04	− 5.57
Midpoint (TSE1)	− 3.31	− 3.09	− 1.59	3.08	− 5.22	− 3.09	− 1.59	− 3.09	− 3.31
Midpoint + 0.2 mm (TSE2)	− 3.11	− 2.89	− 1.39	− 2.88	− 5.02	− 2.89	− 1.39	− 2.89	− 3.11
Midpoint− 0.2 mm (TSE3)	− 3.51	− 3.29	− 1.79	− 3.28	− 5.42	− 3.29	− 1.79	− 3.29	− 3.51

**Figure 21:**
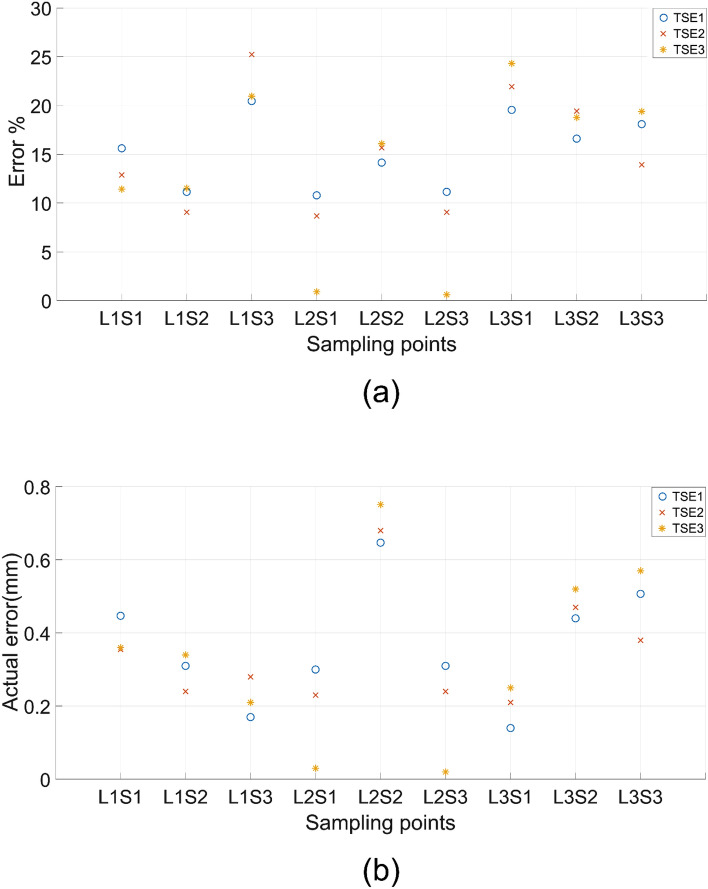
The percentage error (**a**) and the actual error (**b**) between the target set TSE1-TSE3 and the three-point bending test result of the printed specimen.

The differences between the experimental and target deflections at different locations of the cases TSE1 to TSE3 are presented in Fig. 21 both as (a) percentages and (b) absolute values. The maximum deviation between the experimental and target deflections is below 25% or 0.60 mm. Incidentally, the largest percentage error obtained in L1S3 and L3S1 actually have the least absolute deviation at around 0.2 to 0.3 mm, but the percentage error became high as the target deflection values are quite low, less than 2 mm. Apart from these two points, the rest of the results indicate an average of 15% maximum deviations between the experimental and target responses in the deflection profiles of the beam. The FDM print quality is quite variable due to the inherent nature of the deposition of fused polymer could play some role in the loss of convergence. Comparatively, selective laser sintering and melting are likely to offer better control over the meso and microstructures and the consequent deflection responses of the printed beam specimens. Considering all these aspects together with the results presented in Fig. 21, it is pertinent to conclude that the proposed optimisation scheme can effectively alter the deflection profiles of the beams to match with the selected target deflection sets. The outcomes would pave the ways for far better materials and design solutions for compliant mechanisms in future soft robotics and other applications.

## Conclusion

Functionally graded material property responses were achieved by means of computationally altered geometrical forms of voxelised cellular structural forms. The optimisation of geometries of individual cells and the overall responses of the structural continuum were evaluated considering the controlled deflection responses of a simply supported beam with a point load. Three load cases at three specific locations of importance each were considered for deflection responses in combinations to evaluate the effectiveness of the generative search algorithm in converging on the optimised cellular structure of the beam. The classical CMA-ES algorithm is used for the multi-objective optimisation together with the Comsol Multiphysics based finite element evaluations for exploring the generative design space.

Different sets of numerical experiments were conducted to evaluate the performance of the proposed cellular optimisation scheme to match the beam deflection responses with the designated target deflection sets at critical points. The scheme of controlled property variation achieved by varying the cellular geometries based on the optimisation using the CMA-ES algorithm together with the finite element evaluation worked well in achieving the overall objectives of controlling the deflection profiles of simply supported beams with single point loads. Out of the three cellular geometries investigated, the double void geometry was by far the best possible option to achieve an optimised beam structure with less than 5% maximum deviations between the predicted and target deflections at critical points. Experimental validations based on the optimised structures printed by FDM indicate close correlations with numerical predictions, with the average deviations at around a maximum of 15%, in most critical cases. Increased voxel number and degrees of freedom could be the solution for tackling the higher errors in the trails TS4 and TS5. The trends also indicate better results with 40 or more voxels though at higher computational time and cost. However, more advanced technologies such as the computer unified device architecture (CUDA) parallel computing are pathways for better practical utilisation of the outcomes of this research^57^. The proposed optimisation method can be adapted to the laser powder bed fusion (L-PBF) processes with metal materials to further validate the performance of the beams for controlled deflection responses.

## Data Availability

The authors have not used any data outside of the scope of the experimental data already reported in the manuscript. Coding has been generated to integrate the CMA-ES and the Finite Element solutions together, which will be provided later if any user is specifically interested. The datasets used and/or analysed during the current study are available from the corresponding author on reasonable request.
